# Supplementary material for: Are carbon emissions trading and green financial instruments synergistic? -Comprehensive quantitative research based on content analysis
Source: PLoS One. 2024 Mar 7;19(3):e0298601. doi: 10.1371/journal.pone.0298601 (PMC10919601; doi:10.1371/journal.pone.0298601)
Supplement: S1 Data — (ZIP) [file pone.0298601.s001.zip › Data/地级市Z_F工作报告.zip!/╔╜╬≈/┬└┴║╩╨/└┤╫╘╣1⁄2╓┌║┼í╛┬φ┐╦╩2╛▌═°í┐_2019.docx]

　　现在，我代表市人民政府向大会报告工作，请予审议，并请市政协委员和列席人员提出意见。

　　一、2018年工作回顾

　　2018年是全面贯彻党的十九大精神的开局之年。面对错综复杂的外部环境和艰巨繁重的改革发展稳定任务，在市委的坚强领导下，全市上下坚持以习近平新时代中国特色社会主义思想为指导，全面贯彻党的十九大和习近平总书记视察山西重要讲话精神，扎实抓好省委对吕梁提出的“四件大事”，深入实施市委“十大举措”，全面推进三大攻坚战，统筹做好稳增长、促改革、调结构、惠民生、防风险各项工作，经济社会发展稳定性持续性协调性明显增强。

　　全市经济增长同全省一样在由“疲”转“兴”的基础上进一步拓展，全年地区生产总值完成1420.3亿元，增长5.2%，总量较2015年增加将近一半；一般公共预算收入完成174.8亿元，增长25.96%，总量较2015年翻了近一番；进出口总额完成43.7亿元，增长155.2%，增幅全省第一；固定资产投资增长10.3%，社会消费品零售总额增长8%；城乡居民人均可支配收入分别增长6.3%、9.7%。粮食总产量12.27亿公斤，为历史上第二个丰产年。市区优良天数达250天，全省排名第二；秋冬季大气污染综合治理攻坚行动中，市区PM2.5平均浓度值最低、同比降幅最大，在汾渭平原11市排名第一，经济社会发展主要指标取得较好成绩。

　　一年来，我们主要做了以下工作：

　　（一）全力攻坚深度贫困，脱贫攻坚连战连胜。深入实施“3545”年度行动计划，岚县、方山、交口、离石、交城5个县（区）、12.7万贫困人口可望如期摘帽脱贫。健康扶贫、教育扶贫得到国务院副总理孙春兰同志肯定。生态扶贫荣获全国脱贫攻坚组织创新奖，合作化造林模式被国家发改委等3部门联合发文在全国推广。“吕梁山护工”品牌叫响全国，入选全国人社领域精准扶贫20个典型案例。金融扶贫“吕梁模式”走在全国前列，累计发放精准扶贫贷款197.1亿元。708座村级电站和集中式电站并网发电，2.5万户贫困户受益。易地扶贫搬迁87个安置点全部开工，竣工77个，入住7.26万人。县际结对帮扶取得积极成效。351户企业结对帮扶540个贫困村，实施281个扶贫项目。全市广大干部群众和6916名工作队员、1833名第一书记担当作为、砥砺奋进，谱写了新时代“吕梁精神”，涌现出一批又一批“感动吕梁”脱贫攻坚典型人物。

　　（二）加快推进产业结构调整，经济发展迈入转型轨道。大力推进九大产业集群化发展，非煤产业占工业经济比重达到52.4%，工业结构实现反转。孝义金岩250万吨大型焦化等21个焦转化示范项目加快建设。以全省最大的合金铝生产线中润一期43万吨项目通电投产为标志，电解铝生产实现零的突破，铝工业占工业经济比重达10%。白酒产量在2017年增长40%基础上，又实现近20%的较快增长，白酒占工业经济比重达8.6%。“数谷吕梁”建设步伐加快，“一区三园”产业发展体系、“一委三院”人才支撑体系初步建成。旅游收入增长28.4%，全省排名第三。全社会工业用电量181亿千瓦时，增长21.2%，排名全省第一。规模以上工业企业利润增长32.3%，排名全省第一，为2013年以来最好水平。

　　（三）坚持农业农村优先发展，乡村振兴战略稳步实施。持续壮大特色农业产业，打造了孝义、方山、岚县、文水等标准化生产示范县，全市特色产业标准化面积覆盖率达60%以上。实施有机旱作农业“六大工程”，全国首届有机旱作农业科技交流大会在岚县召开。建成兴县2万亩有机谷子、临县5000亩肾型大豆、孝义1500亩绿色有机杂粮等一批示范基地。文水牧标牛业成为全省首家在新三板挂牌的现代畜牧产业企业。连续举办两届名特优功能食品展销会，吕梁特色农产品的品牌效应大幅提升。

　　（四）全面深化改革开放，发展动能加速转换。实施电力体制综合试点改革，吕梁增量配电网初步建成投运。经过不懈努力，以吕梁增量配电网为依托，煤电铝（镁）材一体化发展和改革在省委省政府领导和支持下，正在全力推进、加速突破。推进煤炭供给侧结构性改革，退出煤炭产能60万吨，置换煤炭产能518.8万吨，先进产能占比达到70%。吕梁、岚县、交口3个开发区获批。国有企业“三供一业”分离移交基本完成。PPP公路建设项目融资规模及入库数走在全省前列。企业投资项目承诺制改革扎实推进。出台支持民营经济发展20条措施，用真金白银支持民营企业发展，我市荣获2018年中国区域最佳投资营商环境城市。拓展世界酒文化博览会和大数据等六大招商平台，举办环渤海、长三角、珠三角招商引资暨人才技术合作恳谈会，与中科协等11个中直单位和知名高校签订战略合作协议，聘请72名院士和专家为吕梁转型发展顾问。全年签约项目285个，总投资743.5亿元。柳林、岚县、方山3个县外贸“破零”。首届中国国际进口博览会上，意向成交额全省排名第一。

　　（五）大力加强生态文明建设，三年蓝天保卫战初战告捷。深入开展大气、水、土壤污染防治三大战役，投资近百亿元实施140个生态环保项目。吕梁市区集中供热基本实现全覆盖，新增供热面积495万平方米，12个县（市）城区覆盖率达90%以上。实施汾河流域生态环境综合治理工程，全面开展农村面源污染、黑臭水体和清河专项整治行动，全市主要河流水质优良水体、劣五类水体全部达标。持续深入开展净土专项整治行动。扎实开展环保问题整改“五个回头看”。全省《生态气象监测评估分析报告》显示，吕梁成为全省生态质量改善最为明显的区域。

　　（六）狠抓基础设施建设，城乡面貌明显改善。持续推进市区“双十”重点工程，吕梁政务服务中心、第二污水处理厂一期工程等建成投用，“两带三湖”、图书馆主体基本完工，如意湖公园、城南传统工业主题公园向市民开放，吕梁医疗卫生园区、吕梁一中、社会福利院、体育中心、群艺馆等顺利推进，新区一批遗留问题得到解决，债务和社会风险有效化解。华为大数据中心建成投运，碧桂园落户新区，红星美凯龙建设爱琴海城市综合体等项目达成意向。吕梁市区框架已经拉开，功能日趋完善，产业开始聚集，一座新兴城市正在逐步崛起。推进静兴、祁离2条高速公路建设，实施国道307汾阳段、柳林段等4条国省道改线工程及“黄河一号”旅游公路等项目。建成“四好农村路”2993公里。实施805处农村饮水安全巩固提升工程。开通太原到兴县“蔡家崖”号列车。吕梁火车站发送旅客167.8万人次。吕梁机场吞吐量突破36.5万人次，通航城市达14个。汾阳、岚县、交口国家级园林城市（县城）创建进入省级初验程序。全面铺开农村人居环境三年行动计划，打造了汾阳杏花、离石信义、临县碛口等50个美丽乡村示范点。

　　（七）更加重视社会民生事业发展，人民生活持续改善。全年民生支出达到339.4亿元，占财政总支出的83.1%，增长30.5%。省政府“六件民生实事”圆满完成。实施上安小学、盛地幼儿园等一批市区中小学、幼儿园新建改扩建项目。“教育优质均衡发展的吕梁样本”在《中国教育报》头版刊发。13个县（市、区）定点医疗机构实现先诊疗后付费和“一站式”结算。岚县、临县建成全国健康扶贫示范县，受到国务院扶贫办、国家卫健委通报表彰；交城、孝义公立医院综合改革经验分别被中央改革办、国家卫健委推广。覆盖全民的养老、医疗保险制度体系初步建成。深入开展扫黑除恶专项斗争，共打掉各类涉黑涉恶犯罪团伙97个。全年未发生重特大安全生产事故，各类事故起数与死亡人数实现双下降，人民群众的获得感幸福感安全感不断增强。

　　（八）全面加强政府自身建设，治理能力不断提升。严格落实政府系统全面从严治党主体责任，牢固树立“四个意识”，坚定“四个自信”，坚决做到“两个维护”。自觉接受人大、政协监督，人大代表建议、政协委员提案办复率100%。深入开展“工作落实年”活动，广大干部坚持问题导向，主动担当，亲力亲为，抓重点、攻难点，推动环保攻坚等一批重大任务有效落实。“13710”督办制度深化拓展，政府效能不断提升。支持各民主党派、人民团体工作，国防动员、人民防空、民兵预备役建设进一步加强，粮食供销、外事侨务、文物保护、防震减灾、民族宗教、档案方志、应急管理、对台、气象等工作取得新进步。

　　各位代表，过去的一年，在外部经济环境趋紧的背景下，全市经济社会发展取得积极进展，成绩来之不易。这是习近平新时代中国特色社会主义思想和习近平总书记视察山西重要讲话精神科学指引的结果，是省委、省政府亲切关怀、坚强领导的结果，是市委团结带领全市人民坚持转型发展“三条基本经验”，大干苦干、努力奋斗的结果，是市人大和市政协有效监督、大力支持的结果。在此，我代表市人民政府，向全市人民，向各民主党派、工商联和无党派人士，向各位人大代表、政协委员，向驻吕部队、公安干警，向来吕投资兴业的各位企业家及所有关心支持吕梁改革发展的各界朋友，表示崇高的敬意和衷心的感谢！

　　各位代表，肯定成绩的同时，我们也清醒地认识到，攻坚深度贫困任务仍然较重，临县、兴县、石楼3个深度贫困县贫困发生率仍高于全省平均水平；“一煤独大”结构性问题还未从根本上解决，高新技术和战略性新兴产业占工业经济的比重仅3.7%；城乡居民收入水平滞后于经济发展水平，全省排名靠后；结构性污染治理任务仍然艰巨，以平川四县（市）为重点的区域生态环境质量问题还比较突出；制约高质量发展的体制机制障碍依然存在，营商环境还有待进一步优化；少数干部能力不足、状态不佳、作风不实、担当不够，庸政懒政怠政等现象仍然存在。对此，我们既要直面挑战、敢于担当，又要增强忧患意识，把困难估计的更充分一些，准确把握中央经济工作会议提出的“五个必须”规律性认识，坚定战略自信，保持战略定力，向着全面建成小康社会、实现振兴崛起的宏伟目标迈进！

　　二、2019年工作安排

　　2019年是中华人民共和国成立70周年，是全面建成小康社会的关键之年，是我市在“两转”基础上拓展新局面的攻坚之年。今年政府工作的总体要求是：以习近平新时代中国特色社会主义思想为指导，全面贯彻党的十九大和习近平总书记视察山西重要讲话精神，统筹推进“五位一体”总体布局，协调推进“四个全面”战略布局，按照中央、省委经济工作会议部署和省委对吕梁提出的四项重大要求，在市委坚强领导下，扎实推进“十大举措”，深入实施“改革创新、奋发有为”大讨论，全力攻坚深度贫困，集中力量解决好“两不愁三保障”面临的突出问题；坚持稳中求进工作总基调，坚持新发展理念，坚持推动高质量发展，坚持把供给侧结构性改革和转型综改试验区建设相结合作为经济工作主线，坚持深化市场化改革、扩大高水平开放；以建设示范区、排头兵、新高地“三大目标”为牵引，统筹推进稳增长、促改革、调结构、惠民生、防风险工作；进一步稳就业、稳金融、稳外贸、稳外资、稳投资、稳预期，保持经济运行在合理区间，推动全市经济在由“疲”转“兴”基础上拓展转型发展新局面，为全面建成小康社会收官打下决定性基础，以优异成绩迎接中华人民共和国成立70周年。

　　主要预期目标是：地区生产总值增长6.5%左右，全社会固定资产投资增长7.5%，社会消费品零售总额增长7.5%，一般公共预算收入增长7.3%，城乡居民人均可支配收入分别增长6.5%以上和8%，城镇新增就业4万人，城镇调查失业率、城镇登记失业率分别控制在6%、4%以内。

　　约束性指标是：完成万元GDP综合能耗下降率、万元GDP二氧化碳排放量、万元GDP用水量降幅等省定减排指标任务。市区空气质量持续保持在全省前列。

　　2019年指标设定，是综合各方面因素，顺应内外条件变化，科学合理确定的。地区生产总值增长6.5%，充分估计了当前复杂的经济形势，立足于高质量发展要求，为转型发展留出了空间；固定资产投资、一般公共预算收入增速高于全省平均水平，考虑了一批重大项目支撑，把握了经济运行稳定向好总趋势；城镇居民人均可支配收入增速高于去年，突出了要摆脱全省靠后位置的信心和决心。

　　实现上述目标，必须准确把握国家宏观政策、结构性政策、社会政策等重大政策取向，按照中央“巩固、增强、提升、畅通”八字方针，坚持问题导向、目标导向、需求导向，切实谋划好具有基础性、全局性、牵引性重点工作，推动经济社会高质量发展。

　　今年，我们将重点抓好以下工作：

　　（一）聚焦深度贫困尽锐出战，高质量打赢脱贫攻坚战。

　　进一步聚焦深度贫困县、特殊贫困群体和影响“两不愁三保障”突出问题，坚持不懈落实精准要求，逐县研判、逐项对标、逐个突破，确保临县、兴县、石楼3个深度贫困县全部摘帽、255个贫困村退出、7.7万人脱贫。

　　坚持“一县一策”，攻坚深度贫困堡垒。把扶贫资金、重大项目、政策举措进一步向深度贫困县集中，产业、生态、基础设施、公共服务等项目向深度贫困县布局，“六个帮扶”力量向深度贫困县集结。加大移民搬迁力度，确保4.15万搬迁人口年底前全部入住。用足用活城乡建设用地增减挂钩政策，制定农村废弃、凋敝宅基地及村土地综合整治办法，支持和引导民间资本开发造地。加大退耕还林支持力度，对第一轮退耕还林补助再延补五年。推广金融扶贫“吕梁模式”，组建吕梁农业投资集团，整合市级基金，放大贷款规模，集中支持深度贫困县龙头企业、合作社、村集体经济组织等经营主体。深度贫困县率先启动农村“三变”改革试点，市级对试点村给予资金配套，力争年内覆盖60%的行政村。

　　巩固提升脱贫成效，减少和防止脱贫人口返贫。坚持摘帽不摘责任、不摘政策、不摘帮扶、不摘监管，落实好后续扶持政策，建立完善脱贫成果巩固提升、贫困动态监测、返贫预警等长效机制。对非贫困县非贫困村的贫困人口，统筹用好各类帮扶政策和帮扶资源，确保脱贫不留盲区。坚持问题导向，对国考、省考、脱贫退出第三方评估、省委脱贫攻坚督导等发现问题，建立整改台账，狠抓整改落实，不断提高脱贫攻坚的质量和成色。

　　持续打响脱贫攻坚三大品牌，筑牢织密三张民生保障网。实施“三个100万亩工程”，完成退耕还林和营造林100万亩、经济林提质增效和森林质量提升100万亩、林下经济100万亩，带动2.4万贫困人口脱贫、40万农民增收。深入实施吕梁山护工、技工、工匠培训就业工程，力争就业率达到60%以上。加强光伏扶贫电站运维管理，健全完善收益动态分配机制。持续落实教育扶贫、健康扶贫等政策，筑牢因学因病致贫返贫防线。坚持农村低保和扶贫开发有效衔接，精准动态管理，确保应保尽保、应兜尽兜。

　　各位代表，我市脱贫攻坚已进入滚石上山、爬坡过坎的关键时期，不能有丝毫的放松、停顿、懈怠，必须拿出攻坚拔寨的勇气，一鼓作气，乘势而上，坚决攻克最后堡垒，向历史和人民交出满意答卷！

　　（二）全力推进“深化转型项目建设年”，夯实高质量转型发展基础。

　　持续推进“大项目”战略举措，实施“3331”项目建设大会战，在产业转型、基础设施、社会民生3大领域，实施总投资3600亿元的300多个亿元以上项目，实现大项目顶天立地、小项目开花遍地，力争固定资产投资增速进入全省第一方阵。

　　实施一批重大产业转型项目。围绕煤焦产业高端化发展，实施孝义金岩253万吨7.1米顶装焦化及40万吨乙二醇联产20万吨LNG等19个现代煤化工项目；围绕煤电铝（镁）材一体化发展，实施柳林铝工业园区等9个电解铝及铝材加工项目；以汾阳杏花村开发区为平台，实施汾酒集团机械化酿酒生产线等11个酒类制造、包装、文化旅游等产业延伸项目；以构建“城景通、景景通”旅游公路网为纽带，实施文水苍儿会景区开发等19个文化旅游开发项目；以建设数谷吕梁为目标，实施华为云服务大数据中心二期等10个大数据产业项目；以培育新动能、新增长极为目标，实施孝义远尔州1000万吨低阶煤分质综合利用等74个战略性新兴产业项目。

　　实施一批重大基础设施建设和社会民生项目。围绕完善五纵九横路网框架，实施4条高速、8条国省道改线等项目。加快静兴高速和祁离高速项目建设，争取离石至隰县、汾阳至石楼高速公路年内开工，推进国道209、307和省道340线等8条改线工程。围绕发挥瓦日铁路大动脉作用，实施孝柳铁路至瓦日线连接线和兴县赵家塔等5个集运站建设项目。开展太原—吕梁—延安高铁项目前期工作。运用PPP等模式，实施一批重点水利工程。开工建设北川河改造二期、东川河蓄水改造一期、南川河改造工程；推进县域小水网调蓄水库建设，年内3座调蓄水库完成主体工程，22座完成初设报批工作。

　　全力打通项目建设的堵点痛点难点。牢固树立抓项目就是抓发展的理念，积极落实项目审批、土地调配、征收拆迁、资金保障、水电气暖等要素，确保项目尽早开工。继续推进县级以上领导干部和政府职能部门包联重大项目制度，进工地、到一线、解难题，做到一个项目、一名领导、一套班子、一个方案、一抓到底，推动项目建设掀起新高潮。

　　（三）全面开展制造业振兴升级专项行动，着力打造千亿级以上产业集群。

　　坚决贯彻落实党中央关于实施制造强国的战略部署，培育引进一批十亿级、百亿级龙头企业，着力提升煤焦、铝系加工、钢铁机械制造、白酒、大数据新能源等五大制造业水平，支撑吕梁高质量转型发展。

　　推动煤焦产业向高端化产方向延伸，打造500亿元级现代煤化工产业集群。统筹推进孝义、交城、汾阳和离柳矿区的产能转移、产业整合、项目建设，推动焦化企业向园区集中集聚，向现代煤化工产品高端化方向发展。重点推动孝义福孝化工90万吨煤焦油深加工综合利用等一批焦化转型和现代煤化工项目建设。

　　加快煤电铝（镁）材一体化发展，打造400亿元级铝系产业集群。在省委省政府大力支持下，以吕梁增量配电网改革为突破，积极利用好倒阶梯电价和风电火电打捆等政策，形成增量用电价格洼地，打造全省铝工业集聚区。争取京能临县电厂、孝义晋能电厂入网运行，推动中润一期43万吨合金铝项目全面投产、二期50万吨项目尽快开工，争取柳林阳煤50万吨、交口伊电50万吨铝合金项目尽快落地，带动兴县、孝义、柳林、交口上马一批铝材加工项目。

　　改造提升钢铁、机械制造业，打造300亿元级钢铁机械制造产业集群。加快推进中钢年产200万吨球团及100万吨线材深加工、文水海威产能置换1780高炉及150万吨转炉项目建设。积极推动太钢集团在岚县、临县延伸产业链条，助推钢铁产业改造升级。推动山西晨远铸业公司年产10万吨铸件等项目建成投产。支持交城古特金、文水光华等企业，突破核心基础零部件、先进基础工艺等产业链薄弱环节，提升高端机械制造业配套、加工能力。推动东泰新型特种电缆等一批技改项目投产。

　　大力实施“酒+”融合发展战略，打造200亿元级白酒产业集群。以汾阳杏花村经济开发区为平台，以汾酒集团为龙头，支持晋商酒庄、汾阳王等一批酒类企业发展壮大，推动文水宗酒机械化技改项目投产达效。进一步延伸关联产业，建成投运国家级白酒检测检验中心、白酒交易会展中心及新晋商酒庄四星级酒店，开工建设杏花村特色小镇、汾酒集团五星级酒店，积极策划印象杏花村系列演艺活动，打造集酒类生产、销售、展览、康养和文化旅游为一体的产业集群。举办好第20届（2019）比利时布鲁塞尔国际烈性酒大奖赛和第三届世界酒文化博览会，进一步做大吕梁清香型白酒品牌，提升吕梁产区影响力。

　　围绕大数据、新能源、新材料产业发展，打造100亿元级战略性新兴产业集群。加快建设数谷吕梁。依托军民研究院和华为公司两大品牌，引进一批大数据项目落户吕梁。推动山西中交西部数据中心一期工程等项目建成投运，属陈卫星、天河用友、天河浪潮等一批科技公司落地运营。大力发展新能源产业。加快孝能移动能源产业园项目建设，确保首条生产线300兆瓦铜铟镓硒太阳能电池年内量产出片；推进晋能“互联网+光伏制造业”、华富星辰100兆瓦太阳能电池组件项目建设。积极发展新材料产业。推动利虎玻璃年产150万套汽车安全玻璃生产线、东义镁业年产5万吨高品质镁合金及其制品等项目开工建设，中磁尚善科技公司金属软磁粉芯项目部分投产。

　　各位代表，制造业是现代产业体系的支柱，是转型发展的主战场。吕梁现在的产业总量、发展基础和规划布局，完全有能力支撑1500亿元级以上的五大制造业集群。只要我们持续发力、久久为功，高质量转型发展的目标一定会实现！

　　（四）以文旅融合发展为重点，推进现代服务业加速发展。

　　主动适应消费升级趋势，统筹推动文化、旅游、商贸、物流等服务业发展，把现代服务业打造成经济社会可持续发展的新引擎。

　　努力把文化旅游产业培育成战略性支柱产业。全力抓好旅游项目建设。以黄河旅游板块为重点，完成黄河一号200公里旅游公路主线、38公里支线建设，实现碛口、北武当山、庞泉沟等40个景区景点互联互通。支持交城、岚县、方山创建国家全域旅游示范县。用好奖励政策，支持卦山+玄中寺+英雄广场、三皇庙+孝河湿地公园和杏花村+贾家庄创建国家5A级景区；设立文旅产业基金，抓好碛口等重点景区开发，力争年内实现创建5A级景区和引进文旅战略投资落地“双突破”。大力推动文旅融合发展。坚持文化搭台、旅游唱戏，深度挖掘我市丰富的历史文化、红色文化、廉政文化，将文化资源优势转化为市场竞争优势。持续打造岚县“土豆花开了”、临县“枣儿红了”等旅游文化品牌，举办好吕梁汾阳•贾家庄文学季活动，进一步丰富旅游文化内涵。持续优化旅游服务环境。完善景区停车场、特色餐饮、宾馆等旅游配套设施，加快无线网络全覆盖，推进旅游“厕所革命”，以设施改善带动服务水平提升。打响“吕梁¬——一个令人向往的地方”品牌，引深“乘汾酒号列车，赏吕梁山风光”等旅游季活动，举办旅游专场招商推介会，深入开展旅游形象宣传活动。

　　大力发展商贸物流等现代服务业。以瓦日铁路、太中银铁路为轴线，在离石、柳林、临县、兴县建设集煤炭运输、仓储、洗选、配选、信息咨询等为一体，辐射晋陕蒙等区域的大型现代化煤炭交易市场。以高速公路为依托，在离石、孝义、汾阳建设区域性大型商贸物流园区。整合行业和产业规模，建设临县柳林红枣、汾阳中阳核桃、兴县小杂粮、岚县马铃薯、交口夏菇、文水肉牛等农产品产地集配中心，打造汾阳贾家庄贾街、柳林明清街等一批特色产品市场和商业街区。

　　（五）突出关键领域改革创新，激发转型发展活力。

　　围绕“改革创新、奋发有为”主题，牢固树立“以改革促转型、促民生、促社会治理、促党建、促全面工作”理念，积极落实一批省委、市委作出新部署新要求的重大改革，持续深化一批具有基础性和牵引性的重大改革，巩固提升一批已形成吕梁特色和亮点的重大改革。

　　争当能源革命综合试点“排头兵”。大力推动煤炭产业“减、优、绿”，确保全市煤炭先进产能占比达到75%。年内关闭王家焉、梁家沟等9座煤矿，退出产能750万吨。加快推进晋煤太钢临县三交一号矿井等先进产能项目建设。继续加大现有生产矿井改造力度，新增6座一级安全生产标准化矿井，确保安全高效矿井比重达75%。加快煤层气资源开发利用，实施民用、工业“煤改气”工程，大力推进瓦斯抽取利用项目，年内建成临县嘉浩新能源LNG液化厂、山西中联40万吨LNG液化厂，开工建设兴县华盛燃气LNG项目。实施风电、光伏发电提升工程，积极推进离石云顶山18.45万千瓦、汾阳华电杨家庄10万千瓦、中阳华润二期5万千瓦等项目建设，争取全市风电投产装机规模超过100万千瓦；积极申报交口500兆瓦光伏领跑应用技术基地建设项目。

　　加快开发区改革创新发展。坚持以市场打开发展新通道，运用市场理念谋划开发区发展。持续深化开发区“三化三制”改革，坚持运管分离，向社会公开竞选合法的、有实力的运营机构，积极探索委托中铝公司、汾酒集团等在晋央企、省属国企专业化运营管理和招商引资。建立开发区投融资平台，投资建设开发区道路、供排水、供电、供气等基础设施。支持各开发区与省内外先进开发区、科研院所、高校、大企业等合作共建产业园区。复制推广山西转型综改示范区管理权限，推动市县有关部门、垂直管理部门向开发区派驻机构、人员或向开发区委托授权。

　　稳步推进国资国企改革。在深化混改、专业化重组、分离办社会、国有资本运营上下功夫，实现企业资产重组、优势重塑、业务重构，推动国有企业提质增效。全面完成离柳集团债转股、吕梁中药厂专业化重组为重点的股权多元化改革，统筹解决好省四纺机厂等停产停业企业的遗留问题。

　　积极稳妥防范化解重大风险。全面落实总体国家安全观，坚持“三个坚决防止”和“三个不发生”底线，持续加强防范化解社会、生态环保、安全生产等各领域风险工作。有效防范化解政府债务风险，严控“三公”经费预算，市县两级财政一律按照不低于5%的比例压减一般性支出。积极盘活财政存量资金，提高财政资金使用效率。积极向上争取项目、资金，运用好PPP等融资模式。加强金融风险源头管控，健全风险监测预警处置长效机制。坚决打击各类非法金融机构和非法金融活动，有效防范、打击和处置非法集资，有序推进互联网金融风险专项整治，坚决守住不发生系统性区域性金融风险的底线。积极推进金融体制改革，用好人民银行再贷款等货币政策工具，扩大资金投放规模。调整优化金融体系结构，尽快筹建吕梁地方性银行。扎实推进农信社改制，推动岚县农商行挂牌成立，启动汾阳、方山、交口等7家联社改制工作，力争离石等部分联社获省批筹。

　　大力支持民营企业发展。全面落实省支持民营经济发展30条和我市20条政策措施，充分激发民营企业发展活力。大力支持“个转企、小升规、规改股、股上市”，对首次上规入统、连续2到3年未退出的小微工业企业，给予一定的奖励，年内新发展“小升规”企业30户、“专精特新”小巨人企业2户。积极支持民营企业上市直接融资，对在沪深主板、中小企业板、创业板、科创板上市的企业，在省级200万元奖励的基础上，再奖励200万元；对在全国股转系统挂牌的企业，在省级100万元奖励的基础上，再奖励100万元。落实好市委《关于构建亲清新型政商关系的实施意见》精神，划定“准”与“不准”界线，畅通政商双向沟通渠道，营造支持民营企业家干事创业的良好氛围。继续实施民营企业家“三个一”素质提升工程，组织100名企业家高端培训、1000名企业管理人才管理培训、10000名技术骨干业务培训，努力造就一支勇立潮头的民营企业人才队伍。

　　提升科技创新能力。坚持以创新激发动力活力，大力实施创新驱动发展战略和“百千万人才工程”，出台鼓励创新支持创新的政策举措，促进以观念创新为先导、以科技创新为核心的全面创新。支持企业加大研发投入，对研发经费投入强度全市排名前十位的企业给予奖励，力争年内新增高新技术企业11家、科技型中小企业50户。鼓励企业主动联系对接高校、科研院所，开展校企、专家多元化合作。对创建的重点实验室、工程技术研究中心、科技孵化中心和中试基地，达到国家级、省级、市级标准的，一次性分别奖补500万元、300万元、100万元建设经费。支持离石、孝义、文水、中阳建设省级双创示范基地，交城创建国家首批创新型示范县，柳林建设国家固废综合利用示范基地。建立科技人才引进专项资金，对具体承担我市转型发展重大科研项目、传统产业项目技术革新的“两院”院士、国家“千人计划”专家、国家“万人计划”专家、高技能领军人才和博士研究生，分别给予科研项目资助，争取专家顾问的研发成果、知识产权在吕梁落地生根、开花结果。

　　各位代表，改革关乎国运，创新决胜未来。我们必须以杀出一条血路的精神，争当改革创新先锋，全力打造吕梁的新优势新动力，让人民群众切身感受到改革创新带来的新形象新变化！

　　（六）狠抓主体培育和平台利用，进一步提升吕梁开放型经济水平。

　　围绕破除封闭狭隘，着力解决扩大开放不够问题，整体转身，眼睛向外，积极参与国家重大战略，加大招商引资、招才引智力度，以大开放引领吕梁大发展。

　　积极培育壮大外贸经营主体。制定奖励政策，鼓励企业“走出去”，在研发、生产、销售等方面开展国际化交易合作。运用“互联网+”思维,培育跨境电商主体，力争年内培育100户外贸主体企业，外贸空白县年内全部 “破零”。发挥平川四县（市）的区位和产业优势，继续扩大产品出口；积极支持中部四县（区）利用资源优势开展国际产能合作，推动现代煤化工、钢铁、铝系产品等“走出去”；以红枣、核桃、夏菇、沙棘、小杂粮等为突破口，以标准化建设为引领，推动山上五县优质农特产品走出“国门”。鼓励企业通过技术改造提高出口商品档次和附加值，扩大先进技术、关键设备及零部件进口，组织企业参加第二届中国国际进口博览会，促进全市产业结构优化升级。

　　坚持平台化、品牌化招商引资。进一步拓展招商引资平台。运用已搭建的六大招商平台，一年接着一年办下去，办成具有国内外影响力的大品牌。支持各县（市、区）围绕主导产业编制招商地图，主动对接发达地区、重点园区、知名企业，开展精准招商。各开发区结合自身实际，每年至少主办或承接一次招商引资活动，吸引更多央企国企、世界500强企业、高新技术企业落户吕梁。开展好重大招商引资活动。积极融入“一带一路”建设，举办好京津冀、环渤海、长三角、珠三角、陕甘宁蒙招商引资活动，积极参加广交会、高交会等各类大型展会，吸引更多先进要素汇集吕梁。建立招商项目快速落地综合协调机制，重点跟进去年签约项目，切实提高履约率、落地率。

　　（七）着力提升城市功能与品质，全力打造区域性中心城市。

　　研究制定《吕梁市加快中心城区建设三年行动方案》，坚持主城区提质改造与新区完善功能相结合，坚持市政基础设施、民生和产业项目相结合，坚持财政投资与创新运用金融资本相结合，坚持引进知名品牌企业与动员本地企业投资建设相结合，统筹推进城市规划、建设、管理，不断打造宜居宜业的美丽中心城区。

　　加快行政区划布局调整。积极争取批复柳林撤县设区方案，将中阳、方山按“撤县建区”规划，推动城市空间南北延伸、东西拓展，同步推进市政基础设施建设，同步推进医疗卫生、教育文化设施建设，同步推进商贸物流服务业发展，同步推进吕梁经济技术开发区建设。提前完善“一市四区”城区交通、供热、供气、教育、医疗卫生等功能性基础设施，推动产业链接、服务共享、生态共建，带动城乡区域一体化发展。抓住山西中部盆地城市群一体化发展战略，积极推动平川四县（市）融入太原都市区。

　　全力抓好中心城区建设。坚持一张蓝图绘到底，稳妥有序、滚动实施、压茬推进一批重点项目。以提供优质公共服务为重点，与格盟国际柳林电厂合作引进大热源，改造提升晋能大土河电厂和霍州电厂的供热能力，改造城区老旧管网，实现供热管网互联互通，下大力气解决好市区群众关注的供热“温暖度”问题；年内完工吕梁广场、东川河人行步道、火车站候车大厅提升改造、体育馆和游泳馆维修改造等项目，加快吕梁一中、吕梁高等师范专科学校等一批民生项目建设进度，启动实施城市综合体验馆等项目。以打造优美自然生态环境为重点，开工建设新区东西两山绿化、城南传统工业主题公园二期、凤山底公园、雨污分流管网改造1+N工程等项目。以完善城区路网为重点，推进马茂庄大桥拓宽改造、呈祥路南延三期、新安大道改扩建工程一期、新区路网等项目。以推动住房安居保障为重点，完成离石、方山两县（区）安置房基础设施及配套工程。以提升改善城市风貌为重点，深入开展私搭乱建、占道经营、门头牌匾、坡屋楼顶、建筑立面等拆违治乱集中整治，进一步提升城市整体形象。以产城融合协调发展为重点，争取开工建设吕梁新区航空口岸、吕梁邮政仓储物流基地等项目，推动富力城、红星美凯龙和万达城市综合体项目尽快落地。建设广电中心、科创中心、会展中心、体育中心等一批地标性建筑。建设装备制造产业园、国家数据产业园IDC数据中心二期、晋西北建筑产业园等一批产业项目。

　　大力加强城市管理。进一步推进城市综合执法体制改革，加快推进智慧城市建设，提升城市治理精细化水平。推进农业转移人口市民化，不断提高户籍人口城镇化率。持续推进“五城联创”工作，推动市区、汾阳、岚县、交口创建国家园林城市（县城），交城、汾阳通过国家卫生县城（城市）验收，交口、石楼、方山、兴县申报创建国家卫生县城，柳林、中阳、方山创建省级园林县城。牢固树立“经营城市”的理念，开展中心城区土地收储工作，整合周边零星地块，最大限度发挥收储土地的经济效益和社会效益。

　　各位代表，吕梁中心城区建设是全市人民关注的一件大事，也是市委市政府全力推进的一大工程。随着城市框架拉大，一批重大基础设施项目的加快建设，一批重大民生项目的建成投运，一批产业项目的逐步落地，吕梁中心城区的面貌已越来越清晰地展现在我们面前。我们有决心、有信心、有能力，将吕梁市区打造成蓝绿交织、山水相映、产城融合、宜居宜业的中心城市！

　　（八）围绕全面小康目标，深入实施乡村振兴战略。

　　坚持农业农村优先发展，坚持脱贫攻坚与乡村振兴相结合，统筹抓好“五个振兴”，为全面建成小康社会打下坚实基础。

　　以“一乡一特一园区、一村一品一基地”为载体，在发展特色现代农业上取得新突破。在稳定粮食生产的基础上，做优做强红枣、核桃、小杂粮等特色农产品，打好有机旱作、功能农业、特色优势三张牌，加快推进农业现代化。重点支持100个乡镇建设标准化示范园区，带动1000个村建设生产基地。大力发展有机旱作农业，争取2-3个县列入省级示范县或片区，其他县（市、区）各创建1个千亩示范片区。完成创建国家农业综合标准化示范市目标。以兴县、临县、石楼、汾阳为重点，发展无公害、绿色、有机杂粮，面积稳定在200万亩以上。以岚县、方山、兴县、临县为重点，发展脱毒优质马铃薯100万亩，支持岚县创建省级马铃薯产业园。以交口、临县为重点，建设食用菌产业基地。以方山、石楼、交城、兴县为重点，建设道地中药材规范化种植基地，积极创建省级中药材示范园区。发挥吕粮山猪、牧标牛肉等品牌以及大象集团等龙头企业的带动作用，以交口、文水、中阳、方山为重点，大力发展现代畜牧业，重点打造100个规模健康养殖小区。大力培育新型经营主体，力争年内培育200个农民合作社示范社。持续办好吕梁名特优功能食品展销会，县（市、区）打造各具特色的农产品销售平台，不断扩大特色农产品区域公共品牌影响力。

　　以“五大专项行动”为重点，在农村人居环境整治上取得新突破。按照“示范引领、全面推进”思路，着力实施拆违治乱、垃圾治理、污水治理、厕所革命、卫生乡村“五大专项行动”。按照乡村清洁村、提档升级村、美丽宜居村分类推进，年内打造100个美丽宜居村、200个提档升级村。抓住国家加大基础设施建设的机遇，加快农村道路、饮水、电网等基础设施建设，提升教育、医疗卫生、社会保障、文化体育等公共服务水平。

　　以深化农村改革为动力，在畅通城乡要素合理流动上取得新突破。突出抓好农村基本经营制度、农村土地制度、农村集体产权制度等8项重点改革任务。在全面抓好土地承包期延长、确权登记颁证、流转经营等工作的基础上，加快完成农村集体产权制度改革任务。组建集体经济组织或由村集体入股公司和合作社，统一组织实施基础设施建设和产业项目。扎实开展好“大棚房”问题专项清理整治工作，坚守耕地保护的红线和农地姓农的底线。鼓励支持外出经商务工人员等各类人才返乡创新创业，加强农业科技人才、农村实用人才、农业技能人才队伍建设，不断强化农业农村发展的人才支撑。

　　（九）持续加强生态文明建设，全力打造天蓝地绿、山青水秀的“生态吕梁”。

　　保持铁腕治污的决心和定力不动摇，持续开展环保督察问题“回头看”。以吕梁市区及平川区域，黄河、汾河、三川河、文峪河、磁窑河“两区、五河”为重点，深入实施生态环保“八大工程”，不断提高经济发展的“含金量”“含新量”“含绿量”。

　　以“两区”为重点坚决打赢蓝天保卫战。综合运用“人防、技防、联防”，持续推进“治污、控煤、管车、降尘”和重污染天气应对，全力攻坚大气环境质量突出问题。支持平川4县（市）利用国金、文峰等电厂热源，推动集中供热向周边重点镇、重点村延伸。年内全市完成集中供热清洁改造1050万平方米，清洁采暖改造10万户，城镇既有建筑节能改造200万平方米，农村既有建筑节能改造21万平方米。以市区及周边和汾文交孝为重点，实施区域环境联防联治，制定“一县一策”大气污染防治措施，坚决取缔小散乱污企业，实施工业企业提标改造，持续推进加气站、加油站整治。建立健全重污染天气应对技术体系，建立完善污染物排放应急减排清单和企业“一厂一策”清单，严格落实重污染天气应急响应，确保市区环境空气质量保持在全省和汾渭平原重点城市前列。

　　以“五河”为重点持续打好碧水保卫战。从严落实河长制，坚持饮用水、流域水、地下水、黑臭水、污废水“五水同治”，突出抓好黄河、汾河吕梁段生态修复治理，扎实推进文峪河、三川河、磁窑河等重点河流综合整治，推动流域内企业污水全部达标排放。加快推进9个污水处理厂新建和提质改造，完成汾河、三川河等流域16个城镇污水处理厂和交城经济开发区污水处理厂保（提）温提效改造任务。加快推进市区刘家湾沟、北川河一号坝至交口大桥段2条黑臭水体治理项目，确保城市建成区黑臭水体全部消除。

　　以面源污染整治为重点统筹打好净土保卫战。新建135户规模养殖场畜禽粪污无害化处理设施、3个有机肥厂，确保全市规模养殖场粪污处理全覆盖。大力推进化肥、农药“零增长”行动，完成年度减量任务。深入开展化工行业企业专项整治，着力解决企业危险废物管理不规范、工业固废非法倾倒等问题。开工建设市区生活垃圾焚烧发电和餐厨垃圾处理项目。开展农村环境综合整治，年内9个县（市）全部通过农村生活垃圾治理验收。

　　以建设生态文明为重点扎实抓好生态修复治理。扎实推进汾河流域和3个深度贫困县7条123.7公里的河流生态修复治理。完成水土流失治理70万亩。大力推进吕梁山生态系统修复保护工程，重点打造方山横泉水库水源地保护、交城庞泉沟流域生态修复等10大生态建设项目。继续实施采煤沉陷区综合治理和矿山生态修复示范工程。进一步强化环境污染监管执法，依法严惩重罚生态环境违法行为，持续改善吕梁山生态环境脆弱面貌，以生态文明建设新作为推动转型发展新突破，让蓝天碧水、青山绿岸成为吕梁最鲜明的底色！

　　（十）持续增进民生福祉，切实增强人民群众获得感幸福感安全感。

　　积极回应民生关切，围绕教育、医疗、就业等涉及群众切身利益的问题，加大投入，补齐短板，让吕梁发展更有温度，群众幸福更有质感。

　　坚持教育优先发展战略。加强公立教育，支持民办教育，发展职业教育和幼儿教育。持续加大教育投入力度，早日补齐教育基础设施和教学器材的短板，提高公教人员待遇。积极推进吕梁三中、大武小学等市区10所中小学建设，各县（市）城区根据人口增长幅度，新建一批中小学校和幼儿园，满足进城人员子女入学需求。加强高中校舍和设施建设，满足新高考改革需要。优化整合市直职业教育资源，加快县级职教中心建设，提高职业教育水平。创新教师培养机制，公开招聘一批紧缺学科教师，采取免费教育、学费补偿等方式，统筹推进乡村师范生培养计划。加强城乡校长、教师轮岗交流工作，促进城乡教育均衡发展；继续实施校长、教师综合素质和专业化水平提升计划，进一步提高教育教学质量。加强师德师风建设，规范教师行为和教育秩序。深化市区8所示范校改革，积极推进县管校聘和校长职级制改革，激发教育发展活力，不断提高人民群众对教育的获得感和满意度。

　　补齐医疗卫生事业发展短板。抓好柳林、中阳、兴县等5个县人民医院续建项目，开工建设孝义、文水等5个县中医院和医养中心项目，市医疗卫生园区项目年内主体完工，市人民医院门诊楼10月份前投入使用。深化县域综合医改、城市医联体建设和公立医院改革，抓好市人民医院、汾阳医院和孝义、交口公立医院薪酬制度改革试点。加快推进“互联网+健康医疗”，年内实现县级远程协同医疗全覆盖。逐步更新医疗设备，继续招录一批医疗卫生人才，加大医护人员培训力度。加强中医药服务能力和水平建设。建立医疗、预防、养老整合型健康服务体系，推动以治病为中心向以健康为中心转变。

　　努力健全多层次社会保障体系。深入实施就业优先战略，新增城镇就业4万人，转移农村劳动力4万人。深入实施全民技能提升工程，完成多种职业技能培训8万人。稳妥做好国企职工转岗再就业工作，多渠道解决好国企职工“五险一金”欠缴问题。继续推进农村老年人日间照料中心建设，探索“公建民营”试点工作，支持社会力量发展养老服务业。建立健全服务保障体系，积极落实扶持政策，切实维护退役军人合法权益，提高退役军人服务保障水平。全面落实最低工资标准、提高公职人员工资和离退休人员待遇等政策，努力增加财产性、经营性收入，切实提高城镇居民收入水平。开工棚户区改造项目3094套，基本建成5100套。统筹抓好房地产去库存、控房价、防风险工作，发展住房租赁市场，推动房地产市场平稳健康发展。

　　全面繁荣发展文化广电体育事业。加快图书馆、群艺馆等重点项目建设，改造提升汉画像石博物馆、体育场等场馆。深入推进文明守望工程、乡村文化记忆工程和文化惠民工程，加强碛口等古村镇的保护开发，举办好第四届中国古村镇大会。大力开展全民健身运动，积极承办二青会蹦床、艺术体操比赛项目和2019年全国艺术体操冠军赛及全国青少年游泳U系列锦标赛。精心组织吕梁市第三届运动会。积极打造一批文艺精品品牌赛事，推出一批以脱贫攻坚为题材的文艺作品，完成《晋绥根据地》文献纪录片拍摄，推出民族舞剧《打金枝》，为人民群众提供更多更好的公共文化服务。

　　全力抓好安全稳定工作。牢固树立安全发展理念，突出抓好煤矿、非煤矿山、危险化学品、交通运输、建筑施工等重点行业领域专项整治，强化应急安全管理和应急救援体系建设，以“四铁”要求推动全市安全生产形势持续稳定好转。继续推进“平安吕梁”建设活动，持续深化扫黑除恶专项斗争，加快“雪亮工程”建设，积极防范和依法严惩各类违法犯罪行为，切实维护社会和谐稳定和人民群众生命财产安全。抓好第四次全国经济普查，做实数据、摸清家底。重视国防教育、人防和兵役工作；加强气象、抗震设防、民族宗教、档案方志等工作。支持各民主党派和各人民团体工作。

　　各位代表，今年，我们将围绕群众关心关注的热点难点问题，在完成省政府确定的实施全民技能提升工程、为贫困县农村妇女免费“两癌”检查服务、免费送戏下乡等“八件民生实事”的基础上，集中力量办好我市的“六件民生实事”。一是全市建设55所普惠性幼儿园，其中市区新建改扩建10所、购买普惠性幼儿学位7200个，全市普惠性幼儿园覆盖率达到70%以上；建设55所农村寄宿制学校，确保每个乡（镇）至少有一所寄宿制学校。二是提高城乡低保标准，城市低保标准每人每年提高1200元，农村低保标准每人每年提高600元。三是新建改造“四好农村路”1323公里、贫困村通硬化路511公里。四是建设农村饮水安全工程805处，巩固提升40.9万人的饮水安全。五是启动完成广播电视无线数字化全覆盖工程，确保180万农村人口收看到高清数字电视节目。六是提高深度贫困县农村“两委”干部工资待遇，鼓励广大农村干部在脱贫攻坚主战场上担当作为、勇当先锋。通过切实解决好事关群众切身利益的“头等大事”和“关键小事”，努力让群众的生活一年更比一年好！

　　三、努力建设人民满意政府

　　坚持以政治建设为统领全面推进党的建设，树牢“四个意识”，坚定“四个自信”，坚决做到“两个维护”，自觉用习近平新时代中国特色社会主义思想武装头脑、指导实践、推动工作，着力提高治理体系和治理能力现代化，努力创造全省一流的政务环境。

　　建设法治型政府。维护宪法法律权威，把政府工作全面纳入法治轨道。完成市县政府机构改革，统筹推进承担行政职能的事业单位改革，大幅减少政府对资源的直接配置。依法接受市人大及其常委会的法律监督和工作监督，自觉接受市政协民主监督，主动接受社会舆论监督，广泛听取民主党派、工商联、无党派人士和人民团体意见。全面推进政务公开。严格政府重大事项决策程序，健全法律顾问、公示听证、决策咨询、专家论证等制度，提高政府决策科学化、民主化、法治化水平。

　　建设服务型政府。继续深化企业投资项目承诺制改革，探索推进市县相对集中行政许可权改革试点。巩固深化“3545”专项改革，聚焦企业开办、不动产登记、项目审批等重点领域，进一步简环节、减材料、优流程、压时限、提速度。加快构建市县乡一体化网上政务服务平台，推行政务服务“网上办理”，实现数据“多跑路”、企业和群众“少跑腿”甚至“不跑腿”。严厉打击强揽工程、强供地材、无理阻工等干扰破坏企业生产经营秩序的行为，为企业依法经营、合规发展提供有力支持。

　　建设担当型政府。认真开展“改革创新、奋发有为”大讨论，按照省委“六个破除”“六个着力”“六个坚持”部署要求，做好10个关键环节的规定动作，推动思想再解放、改革再深入、创新再发力、开放再提质、工作再抓实，在落实一批重点工作、推进一批重大改革、选树一批先进典型上取得新成效。落实容错纠错机制，切实为敢于担当的干部撑腰鼓劲。树立担当为荣、避事为耻的理念，各级干部都要担在关键、干在实处、走在前列，既出力又出彩。提倡“一线工作法”，人要沉下去，事要扛起来，打造一支忠诚干净担当、改革创新有为的干部队伍。以大讨论为契机，全体公务人员都要主动对标、积极超标、敢于立标，树一流标准、练一流本领、创一流业绩，推动更多工作进入全国、全省第一方阵，努力创造更多走在前列的“吕梁模式”。

　　建设廉洁型政府。切实扛牢全面从严治党主体责任，持续推进政府系统党风廉政建设和反腐败斗争。深入贯彻中央八项规定精神及省委、市委实施细则，坚决反对形式主义、官僚主义。认真履行“一岗双责”，切实抓好中央、省委、市委巡视巡察和经济责任审计反馈问题整改，持续加强重点领域、关键环节权力运行制约监督，推进审计监督全覆盖。用好“13710”等督查督办手段，完善考核奖惩激励机制，狠抓工作落实。坚决惩治各类腐败行为，让清正廉洁成为干部的自觉追求。

　　各位代表，新时代催人奋进，新使命更需实干。让我们更加紧密地团结在以习近平同志为核心的党中央周围，在市委的坚强领导下，改革创新，锐意进取，奋力谱写吕梁脱贫攻坚、转型发展新篇章，以优异成绩迎接中华人民共和国成立70周年！
